# Submucosal urethral bulking with 2.5% polyacrylamide hydrogel (Mictamid) for urinary incontinence in male dogs using a percutaneous fluoroscopically assisted perineal approach

**DOI:** 10.1093/jvimsj/aalag140

**Published:** 2026-07-17

**Authors:** Rae McAtee, Stephanie M Skinner, Jonathan D Foster

**Affiliations:** Nephrology Urology Service, Friendship Hospital for Animals, 5025 Wisconsin Ave NW, Washington, DC 20016, United States; Nephrology Urology Service, Friendship Hospital for Animals, 5025 Wisconsin Ave NW, Washington, DC 20016, United States; Nephrology Urology Service, Friendship Hospital for Animals, 5025 Wisconsin Ave NW, Washington, DC 20016, United States

**Keywords:** canine, incontinence, Mictamid, urethral bulking, USMI

## Abstract

**Background:**

Urethral bulking using cystoscopic injection of various bulking agents has been commonly utilized in female dogs with urethral sphincter mechanism incompetence (USMI), but its use in male dogs with urinary incontinence has not been well described.

**Hypothesis/Objectives:**

Describe the treatment and outcomes of male dogs with urinary incontinence that underwent submucosal cystoscopic injection of 2.5% polyacrylamide hydrogel (PAHG) using a percutaneous fluoroscopically assisted perineal approach.

**Animals:**

Five client-owned male dogs with urinary incontinence.

**Methods:**

Retrospective case series. Male dogs with urinary incontinence that underwent a percutaneous fluoroscopically assisted perineal approach for submucosal cystoscopic injection of PAHG. Complications were recorded. Continence scores (1 = constant incontinence to 5 = fully continent) were assessed before the procedure, 30 days after bulking, and at the time of last follow-up and compared using a Friedman multiple comparison test.

**Results:**

All 5 dogs had suspected USMI and 1 dog also had a urethral diverticulum. All 5 dogs had improvement in their urinary incontinence. The median initial continence score was 2 (range, 2-4). The continence score improved significantly compared with the baseline score both at 30 days post-bulking (median, 5; range, 4-5) and at the time of last follow-up (median, 5; range, 4-5; *P* = .03). Median time of last follow-up was 108 days post-bulking (range, 51-154 days). One dog had self-limiting hematuria. No severe adverse events were observed.

**Conclusions and Clinical importance:**

Percutaneous fluoroscopically assisted perineal approach for submucosal cystoscopic injection of PAHG in male dogs with urinary incontinence is a promising treatment option.

## Introduction

Urethral sphincter mechanism incompetence (USMI) is a common cause of acquired urinary incontinence in female dogs. Medical management can be successful with either phenylpropanolamine or estrogen administration or both. In female dogs with suspected USMI, 85.7% were reported to be fully continent after receiving phenylpropanolamine at a dosage of 1 mg/kg q8h.^1^ Additional studies have shown that dosages as low as 1.5 mg/kg of phenylpropanolamine q24h can be effective to control urinary incontinence in up to 88% of female dogs.^2^ Another study showed 83% of female dogs became either fully continent or had improved incontinence after administration of different estrogens at variable dosages.^3^^,^^4^ In affected female dogs that are medically unresponsive, urethral bulking or an artificial urethral sphincter (AUS) are considered the next recommended treatment options. Urethral bulking performed using cross-linked gelatin in female dogs resulted in over 80% of these dogs achieving continence.^5^ Urinary incontinence is observed less frequently in male dogs than in female dogs, with an estimated prevalence of 1%.^6^ Estrogen treatment is not indicated in male dogs, with only 43% of male dogs showing a good or excellent response.^7^ In male dogs that do not respond to phenylpropanolamine, IM injections of testosterone cypionate every 4 weeks have been utilized, but in a study of 8 male dogs only 3 had a good to excellent response.^8^

Because many male dogs with USMI do not respond to medical treatment, alternative management options are needed. In a previous study, 4 male dogs with USMI underwent urethral bulking using polytetrafluoroethylene (Teflon), and performed using a laparotomy approach with all 4 dogs achieving continence for a duration of 2-28 months.^9^ Surgical AUS placement resulted in short-term continence in 84% of dogs and improved long-term continence in 60%.^10^ Both of these options involve laparotomy, and therefore less invasive techniques are needed to achieve continence in male dogs.

The purpose of our retrospective case series is to describe a percutaneous fluoroscopically assisted perineal approach for performing cystoscopic urethral bulking in male dogs with urinary incontinence secondary to USMI.

## Materials and methods

### Study population

The medical record system from Friendship Hospital for Animals was searched between January 2025 and July 2025 using the keywords “bulking,” “perineal access,” and “2.5% iPAAG.” Dogs were included if they were males, had urinary incontinence, underwent the percutaneous fluoroscopically assisted perineal approach with endoscopic urethral bulking, and had at least 1 follow-up communication with the owner.

### Procedures

Dogs were anesthetized, intubated, and maintained under general anesthesia throughout the procedure. Anesthesia protocols were at the anesthesiologist’s discretion. At the discretion of the attending clinician, dogs received cefazolin 30 mg/kg q90 min throughout the procedure. The procedure included flexible cystoscopy followed by fluoroscopically assisted perineal access to allow for rigid cystoscopy and subsequent urethral bulking. The full procedure is described in the Appendix. After the urethral bulking was performed, the bladder was emptied using manual expression. The dogs recovered from general anesthesia and were discharged the same day.

### Follow-up

Owners were contacted and asked to score their dogs’ continence with a previously utilized 5-point scoring system.^11^

1: The patient is constantly incontinent. Will leak urine when awake as well as when asleep.

2: The patient is poorly continent and will intermittently leak urine.

3: The patient occasionally leaks urine, more commonly when asleep than when awake.

4: The patient is mostly continent and only leaks urine sometimes when sleeping.

5: The patient is always continent.

Owners provided a urinary continence score before the procedure, 1 month after the procedure, and at their last communication post-procedure. Owners also were asked to describe any complications noted after the procedure as well as how satisfied they were with the procedure.

### Statistics

Pre-procedure continence scores were compared to 1-month post-procedure scores as well as the continence scores at the time of last follow-up using a Friedman multiple comparison test. A *P*-value < .05 was considered significant.

## Results

### Study population

Eight male dogs were identified from the medical record review. Three cases were excluded because urethral bulking was discussed in the medical record but not performed. The remaining 5 cases were included. Breeds included mixed breed dogs (*n* = 3), Spanish Water Dog (*n* = 1), and Cane Corso (*n* = 1). Dogs were 2-11 years old (median, 6.5 years) at the time of the procedure and weighed 18.9-68.6 kg (median, 35.6 kg). All 5 dogs were previously neutered.

Physical examination and neurologic examination did not identify any abnormalities. All dogs had baseline blood testing before their procedures. One dog had a static, historically noted cholestatic hepatopathy. It was suspected to be secondary to previously diagnosed atypical hypoadrenocorticism 5 years prior that was managed with 0.05 mg/kg prednisone daily. One dog had stable stage II chronic kidney disease. No other abnormalities were noted. Two dogs had normal urinalysis results on urine obtained by cystocentesis with no evidence of pyuria or bacteriuria. Two dogs had subjectively normal post-voiding urine residual volumes. Four dogs had abdominal ultrasonography that did not identify any abnormalities of the urogenital tract.

One dog had been hit by a car a year before initial evaluation for urinary incontinence and had undergone multiple surgical interventions that included right caudal abdominal traumatic hernia repair, closed right craniodorsal coxofemoral luxation, toggle pin fixation with placement of an internal Ehmer trochanteric ilial suture, and left sacroiliac luxation repair using lag screw placement. This dog did not experience urinary incontinence until after the traumatic event. One dog had a history of a urinary tract infection 7 months before initial evaluation as well as intermittent glucosuria with a normal blood glucose concentration and normal urine sediment.

The median continence score before the procedure was 2 (range, 2-4) with a median duration of incontinence before the procedure of 1 year (range, 1-4 years). Three of the 5 dogs were receiving immediate-release phenylpropanolamine at the time of initial evaluation. Two dogs were on twice daily dosing with 1 at 1.4 mg/kg and the other at 2.6 mg/kg. The dog on 1.4 mg/kg q12h showed resolution of its urinary incontinence on phenylpropanolamine for 22 months, but incontinence returned despite continued phenylpropanolamine use and therefore urethral bulking was elected. The third dog was receiving 1.4 mg/kg phenylpropanolamine twice a day and an additional mid-day dose of 0.7 mg/kg. This dog showed improvement with phenylpropanolamine with resolution of urine leakage when awake, but urine leakage continued at night. One dog initially received the extended-release formulation at 4 mg/kg once a day, then was changed to immediate release at 2.2 mg/kg twice a day without improvement. One dog did not receive any medical management before urethral bulking was pursued.

### Procedure

Patients were under anesthesia for a median of 105 min (range, 90-160 min). Four of the 5 dogs had no abnormalities identified on cystoscopic evaluation, and 1 dog had an approximately 6 *×* 8 mm proximal urethral diverticulum. Three dogs had fluoroscopic contrast cystourethrograms performed. One dog had a normal contrast study, 1 dog had a mild intrapelvic bladder based on the location of the bladder trigone within the pelvic girdle upon complete filling, and 1 dog had a small urethral diverticulum identified in the most proximal part of the urethra, consistent with cystoscopic findings. The urethral bulking in this dog was performed caudal to the urethral diverticulum.

**Figure 1:**
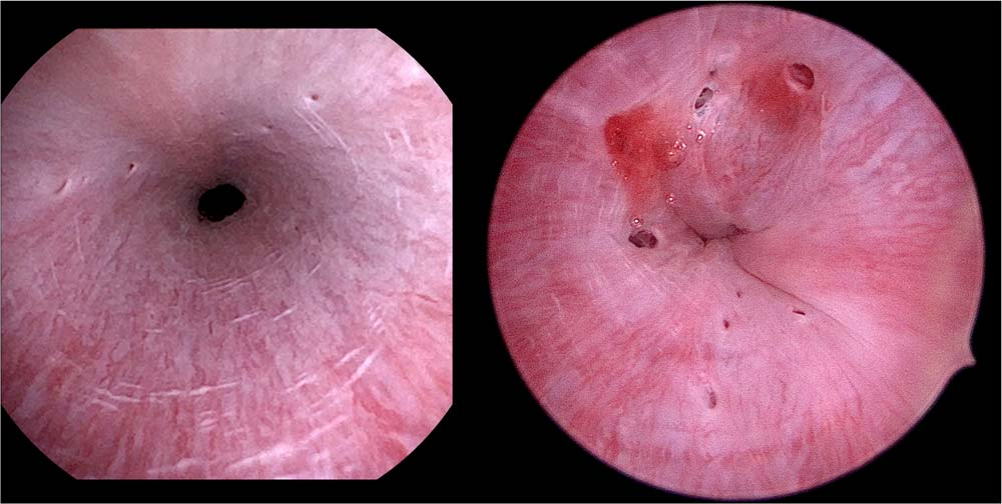
Cystoscopic images pre (left side) and post (right side) submucosal injections with PAHG. Abbreviation: PAHG = polyacrylamide hydrogel.

All dogs had 2 rings of PAHG injected into their urethras (Figure 1). A total of 2.5% PAHG is homogenous, requires no mixing, and does not change in consistency throughout the injection process. The needle was immediately removed after injection and clear hydrogel could be seen in the submucosal cushion through the needle hole with no significant product leakage from the injection site around the needle or after the needle was withdrawn. A median of 5.5 mL of PAHG was utilized during the procedure (range, 4-6 mL) with 1 dog not having documentation of how much volume was utilized.

### Follow-up and complications

One dog had hematuria noted for a few days post-procedure, which resolved without intervention. No other complications were reported. Notably, no dogs developed clinical signs of urinary retention or urinary tract infection after the procedure.

**Figure 2:**
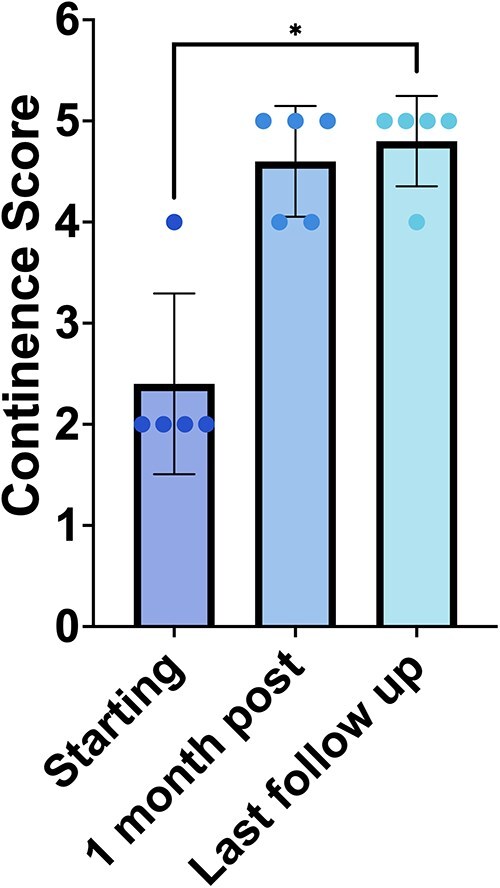
Continence scores before submucosal urethral bulking, 1 month post-submucosal urethral bulking, and at the point of longest follow-up (range, 51-154 days). The continence score at the longest point of follow-up was significantly higher compared to pre-procedure scores (*P*-value .03).

The median continence score before the procedure was 2 (range, 2-4). The median continence score 1 month post-procedure was 5 (range, 4-5). The median time of last follow-up was 108 days post-bulking (range, 51-154 days). At this time the median continence score was 5 (range, 4-5), which was significantly higher compared with pre-procedure scores (*P* = .03) (Figure 2). Four dogs achieved continence scores of 5 (completely continent) at their longest point of follow-up, including the dog with the urethral diverticulum. One dog had a continence score of 4 at 1 month post-procedure and thereafter. This dog was the one that remained on phenylpropanolamine at the same original dose as used before bulking. No other dogs had phenylpropanolamine continued post-procedure. No dog had a decrease in continence score between the 1-month scoring and the longest point of follow-up. All owners reported being satisfied with the outcome of the procedure.

## Discussion

With historically low response rates to medical management for USMI reported in male dogs, it is important to investigate other treatment modalities.^7^ Our case series demonstrates that male dogs with urinary incontinence presumably secondary to USMI that fail medical management can be treated successfully and in a minimally invasive manner using cystoscopic urethral bulking. The percutaneous fluoroscopically assisted perineal approach allowed for bulking using a rigid cystoscope, because no available needles allow smooth injection of a viscous bulking agent through a flexible ureteroscope. One of the dogs also had a small urethral diverticulum, indicating that urethral bulking caudal to the diverticulum also may be a viable treatment option for this condition as well. Although none of the dogs had a urethral pressure profile performed to confirm USMI, structural abnormalities were ruled out in the 4 dogs without a urethral diverticulum, and the incontinence pattern was most consistent with USMI. All 5 dogs in our case series showed improvement or resolution in their incontinence at home in the short-term (up to 5 months) with longer-term data still being needed.

Medical management for USMI in male dogs can include administration of phenylpropanolamine with good response rates reported in only 43% of cases (7/16 male dogs) or testosterone cypionate, but low response rates for testosterone administration have been reported with only 38% (3/8) of dogs having a good response.^7^^,^^8^ Historically, male dogs with an inadequate response to medical management of urinary incontinence had few options for further treatment beyond surgical placement of an AUS or urethral bulking performed antegradely using a surgical approach through the bladder.^9^^,^^10^ Prostatopexy also has been evaluated in a small case series with only 1/9 dogs having complete resolution of incontinence, and 4/9 showing only a partial response.^12^

Placement of an AUS improved short-term incontinence (<12 months) in 84% of affected male dogs and long-term incontinence (>12 months) in 60%. Minor complications included temporary stranguria, inflammation at the port, or hematoma in 4/16 cases (25%). Major complications included mechanical obstruction leading to stranguria, fistula formation, or cuff rotation in 5/16 cases (31%), with 4 of these dogs (25%) requiring surgical revision.^10^ An additional potential major complication includes leakage of the cuff, which was seen previously in a female dog with an AUS placed.^13^ Additionally, the development of urethral obstruction secondary to fibrous capsule formation around the AUS has been reported 20-2457 days after placement, and required removal of the device.^14^ Minor complications include development of urinary tract infection after AUS placement, which has been reported in up to 40% of affected dogs.^15^

Previous reports of urethral bulking in female dogs have shown that complications can include short-term and self-limiting stranguria, hematuria, or pollakiuria.^5^^,^^16^^,^^17^ In male dogs that have undergone perineal access, complications have included leakage of urine from the perineal access site for up to 48 h after the procedure in 16% of cases, stranguria in 5% of cases, and mild perineal irritation in 5% of cases.^18^ All of these complications were self-limiting and resolved within several days. Another study utilizing percutaneous perineal access for ectopic ureteral laser ablation showed that 1/18 male dogs (5%) experienced dysuria that resolved with a short course of tamsulosin.^19^ These dogs ranged in weight from 6.6 to 66.4 kg.^19^ This study demonstrates that perineal access can be performed in most male dogs, but size can be a limiting factor. The pelvic urethra needs to accommodate a peel away sheath large enough for the cystoscope to pass. We used a 16 Fr sheath to accommodate our cystoscope, but narrower sheath and scope combinations can be used in smaller dogs. The smallest dog in our study was 18.9 kg, and no alterations to accommodate a smaller dog were required. Two male dogs that underwent perineal access with dilatation of the urethra up to 26 French showed no urethral abnormalities on necropsy examination 2 weeks later.^20^ Urethral obstruction has not been reported as a complication secondary to submucosal urethral bulking in dogs.

The main limitation with urethral bulking is the potential need for repeat bulking in the future to maintain continence. Re-bulking may be required if the injected bulking material is resorbed or if the cushions created in the submucosa have ruptured.^17^ In female dogs that were bulked with glutaraldehyde cross-linked collagen, 71% of cases (17/24 dogs) had improvement in their incontinence and the average duration was 45 months. Submucosal bulking using dextranomer hyaluronic acid was successful in 58% (15/26) of female dogs, and those dogs remained continent for an average of 21 months.^16^ With bovine cross-linked collagen injections, 68% of dogs (27/40) responded, with an average duration of 17 months post-injection (range, 1-64 months) with 40% showing deterioration in results over time.^17^ Cross-linked gelatin submucosal injections improved incontinence in 91% (20/22) of female dogs, with a mean duration for continence of 11 months.^5^

The bulking agent used in our study was 2.5% PAHG, a 2.5% cross-linked polyacrylamide in 97.5% water. It has been administered intraarticularly in horses and dogs for managing osteoarthritis.^21^^,^^22^ It also has been shown to be safe in a multicenter study evaluating urethral bulking in 135 women with the most common adverse event being urinary tract infection, seen in 10 women. Other adverse effects included injection site pain in 5 women, urinary retention in 2 women, hematuria in 2 women, urge incontinence in 4 women, injection site laceration in 1 woman, and headaches in 4 women.^23^ A study that evaluated 171 women with stress urinary incontinence that underwent PAHG urethral injections, showed that treatment success occurred in 60% of women at less than 1 year and in 53% of women 7-8 years after initial injection.^24^ Of these women with successful outcomes, 58% underwent 2 injection procedures and 42% had a single injection procedure. Another study evaluating 388 women that had urethral bulking using PAHG showed that 65% of women (253/388) felt cured or improved 7 years after the procedure.^25^ Additional follow-up research is needed to determine if the duration of continence observed in dogs is similar to what is observed in women treated using PAHG bulking.

One male dog in our study had suspected acquired urinary incontinence from trauma after being hit by a car and having multiple orthopedic surgeries. This dog’s fluoroscopic contrast study during his cystoscopy showed the presence of a urethral diverticulum in the proximal urethra. Previous studies have reported some success with surgical correction of the diverticula or with placement of AUS to control the incontinence.^26^^,^^27^ The dog treated in our study illustrates that urethral bulking caudal to the diverticulum represents an additional therapeutic option for managing urinary incontinence in male dogs with urethral diverticula.

Our case series had several limitations. The main limitation is the retrospective nature and lack of standardization in pre-procedural diagnostic evaluation and management. Only 4 of the 5 dogs included in our case series were treated with phenylpropanolamine and no dogs included were given testosterone cypionate before pursuing urethral bulking. Therefore, it is unknown if all dogs had fully failed medical management for their incontinence. The diagnosis of USMI was presumptive, because urethral pressure profiling was not available. Additionally, long-term follow-up was not available beyond 5 months (median time, 3.5 months). Another limitation for widespread adoption of this technique is the use of a fluoroscopic approach for perineal access. A non-fluoroscopic method of obtaining access includes palpation of the urethra with the aid of a urinary catheter and saline boluses.^17^ No other methods of access are currently available in the literature for comparison. Despite its limitations, our case series indicates that a percutaneous fluoroscopically assisted perineal approach for submucosal cystoscopic urethral bulking with PAHG is a tenable treatment option for male dogs with USMI.

## Off-label antimicrobial declaration

The authors declare no off-label use of antimicrobials.

## Institutional animal care and use committee or other approval declaration

The authors declare no institutional animal care and use committee or other approval was needed.

## Appendix

### Procedures

Dogs were positioned in dorsal recumbency with legs in a frog leg position. The peri-preputial and perineal hair was clipped, and the skin was aseptically prepared. Patients were draped and an 8.5 Fr flexible ureteroscope (Flex XC, Storz, El Segundo, CA) was introduced into the urethra under continuous saline irrigation for evaluation of the urethral mucosa, bladder mucosa, and identification of the ureterovesical junctions (UVJ). The flexible ureteroscope then was removed and in some cases a contrast cystourethrogram was performed by passing an 8 Fr polyvinyl catheter into the urethra and filling the bladder and urethra with a 1:1 mixture of iohexol:saline (0.9% NaCl) under fluoroscopic observation.

A perineal approach was performed as previously described, so that a 16 Fr 15.5 cm length peel away sheath (Peel-Away Sheath Introducer, Cook Medical, Bloomington, IN) was positioned transcutaneously with the tip of the sheath in the urinary bladder.^18^ A 2.9 mm *×* 30 cm rigid 0-degree telescope with a 15 Fr sheath and a 5 Fr working channel (Karl Storz, Tuttlingen, Germany) was passed through the sheath and utilized for the remainder of the procedure. The scope and sheath were positioned in the proximal urethra. A 23-gauge 35 cm Williams cystoscopic injection needle (Cook Medical, Bloomington, IN) was passed through the working channel of the cystoscope and the 2.5% polyacrylamide hydrogel (PAHG) syringe (Mictamid, ConturaVet, Franklin TN) was attached to the needle. Each syringe contained 1 mL of 2.5% PAHG. The syringes were stored at room temperature, and no mixing or other specific preparation of the material was done before injection. With cystoscopic observation, the needle was directed into the submucosa of the proximal prostatic urethra with the bevel pointed toward the lumen. Under slow continuous saline irrigation to maintain visualization, PAHG was injected to produce a submucosal cushion and elevation of the mucosa toward the center of the urethral lumen. This procedure was performed 4-5 times in a circumferential arrangement until > 75% urethral coaptation was visualized. The procedure then was performed a second time approximately 2 cm distal to the first ring. The scope and sheath were removed, and the perineal incision was left to heal by second intention.

## Abbreviations

AUS: artificial urethral sphincter
PAHG: polyacrylamide hydrogel
USMI: urethral sphincter mechanism incompetence
UVJ: ureterovesicular junction
